# Mortality and morbidity in children caused by falling televisions: a retrospective analysis of 71 cases

**DOI:** 10.1186/1865-1380-4-11

**Published:** 2011-03-22

**Authors:** Servan Gokhan, Ozkan Kose, Ayhan Ozhasenekler, Murat Orak, Mehmet Ustundag, Cahfer Guloglu

**Affiliations:** 1 Accident and Emergency Department, Diyarbakir Education and Research Hospital, Diyarbakir, Turkey; 2 Orthopaedics and Traumatology Clinic, Diyarbakir State Hospital, Diyarbakir, Turkey; 3 Emergency Clinic, Diyarbakir State Hospital, Diyarbakir, Turkey; 4 Department of Emergency Medicine, Dicle University School of Medicine, Diyarbakir, Turkey

**Keywords:** Wound and injuries, television, home accidents, child

## Abstract

**Objectives:**

To quantify injuries in children that result from toppled televisions.

**Methods:**

Children presenting directly to emergency department due to injuries caused by falling televisions were identified from our digital patient database, and a retrospective chart review of 71 children was performed. Descriptive statistics were applied.

**Results:**

71(1.8%) out of 3856 admissions due to injuries sustained at home were TV-related injuries. There were 50 (70.4%) boys and 21(29.6%) girls. Mean age was 39.79 ± 20.14 SD months. Almost three quarters of the children (49/71) sustained various head and facial injuries. There was traumatic brain injury in 14 patients, extremity injuries in 30 patients, thoracic injuries in 13 patients and abdominal injuries in ten patients. 16 patients were hospitalized. 14 of them required follow-up in intensive care unit. Two patients (one with epidural hematoma and one with subdural hematoma) underwent surgical intervention. Four patients with subarachnoid bleeding died. The mean length of hospital stay was 71.25 hours (range, 48-168) in hospitalised patients. The overall mortality rate was 5.6%.

**Conclusions:**

Falling TVs may cause significant morbidity and mortality in children particularly those younger than 3 years old. Head and facial injuries are the most common body region involved and traumatic brain injury is the major cause of death.

## Correction

Owing to an unfortunate mistake in typesetting, the abstract of this article [1] was deleted and replaced with the abstract of an unrelated article. The correct abstract is reproduced here. The publisher deeply regrets this error.
